# Silica-based microencapsulation used in topical dermatologic applications

**DOI:** 10.1007/s00403-023-02725-z

**Published:** 2023-10-04

**Authors:** Lawrence J. Green, Neal D. Bhatia, Ofer Toledano, Maya Erlich, Amy Spizuoco, Benjamin C. Goodyear, Jean Philippe York, Jeannette Jakus

**Affiliations:** 1 George Washington University School of Medicine, Washington, DC USA; 2 Therapeutics Clinical Research, San Diego, CA USA; 3 Sol-Gel Technologies, Ness Ziona, Israel; 4 True Dermatology PLLC, New York, NY USA; 5 Galderma Laboratories, L.P., Fort Worth, TX USA; 6 SUNY Downstate Health Sciences University, Brooklyn, NY USA

**Keywords:** Microencapsulation, Dermatologic agents, Dermatological agents, Silicon compounds, Amorphous silica

## Abstract

Microencapsulation has received extensive attention because of its various applications. Since its inception in the 1940s, this technology has been used across several areas, including the chemical, food, and pharmaceutical industries. Over-the-counter skin products often contain ingredients that readily and unevenly degrade upon contact with the skin. Enclosing these substances within a silica shell can enhance their stability and better regulate their delivery onto and into the skin. Silica microencapsulation uses silica as the matrix material into which ingredients can be embedded to form microcapsules. The FDA recognizes amorphous silica as a safe inorganic excipient and recently approved two new topical therapies for the treatment of rosacea and acne. The first approved formulation uses a novel silica-based controlled vehicle delivery technology to improve the stability of two active ingredients that are normally not able to be used in the same formulation due to potential instability and drug degradation. The formulation contains 3.0% benzoyl peroxide (BPO) and 0.1% tretinoin topical cream to treat acne vulgaris in adults and pediatric patients. The second formulation contains silica microencapsulated 5.0% BPO topical cream to treat inflammatory rosacea lesions in adults. Both formulations use the same amorphous silica sol–gel microencapsulation technology to improve formulation stability and skin compatibility parameters.

## Introduction

The successful delivery of topical drugs and/or excipients can be influenced by various internal and external factors, including drug concentration [1], drug potency [1], oxidation [2], UV light exposure [3], physicochemical factors (drug molecule weight, lipophilicity, pH, size) [4], and the interaction and compatibility with other ingredients (i.e., combination products containing tretinoin and benzoyl peroxide) [5]. Encapsulating these ingredients in silica can improve their stability and control their release onto and into the skin [6, 7].

Since its introduction in the 1940s, microencapsulation has received widespread attention because of its diverse capabilities. Microencapsulation is the process of entrapping a microsized active ingredient particle (core material) within a shell (shell/wall material) [8]. Microencapsulation technologies are commonly used to provide cosmetically elegant [9] and nontoxic methods to protect, direct, and control the release of active ingredients [10], leading to improved stability, efficacy, and patient adherence [11]. Microencapsulation can also enhance the sensory properties of cosmetics, giving the product a more elegant look and feel [12]. In addition to protecting and stabilizing bioactive compounds [13], microencapsulation allows manufacturers to minimize medication doses while maintaining efficacy and reducing adverse effects [14].

The two most common microencapsulation techniques are chemical and physical, which can be further categorized into physicochemical and physicomechanical subtypes [15]. Silica-based microcapsules can be formed using the sol–gel process. In this process, amorphous silica is formed by interconnecting colloidal particles (the “sol”) under increasing viscosity until a rigid network, the silica shell (the “gel”), is formed [5]. Tetraalkoxysilanes undergo hydrolysis and polycondensation reactions to form amorphous silica [16]. This method results in sol–gel microcapsules with several valuable properties. The microcapsules range from 0.01 to 100 µm [17]. The solid form of the active ingredient [e.g., benzoyl peroxide (BPO) or tretinoin (all-trans retinoic acid)] functions as the core during the sol–gel reaction, and a silica shell forms around it [18].

Amorphous silica is listed in the inactive ingredient guide of the United States Food and Drug Administration (FDA) [17]. Excipients or inactive ingredients are a crucial component of drug formulations that are added intentionally to aid in the manufacturing process or to enhance the performance, stability, bioavailability, or acceptability of the topical drug product [19]. These substances are generally inert or nonreactive and include emollients, emulsifiers, gelling agents, surfactants, preservatives, buffering agents, or solvents [20, 21]. Excipients are considered safe and typically do not directly interfere with the therapeutic action of the drug [19, 22]. The use of excipients is essential in the formulation and delivery of pharmaceutical products, as they can impact the pharmacologic properties of the drug [22], as well as its appearance, color, odor, sensory properties, and shelf-life stability [23]. In this case, amorphous silica has no direct effect on the treatment of disease and exerts no effect on any structure or function of the human body [24]. In addition, silica is compatible with various active pharmaceutical ingredients (APIs), making it particularly useful in drug development [17].

**Fig. 1:**
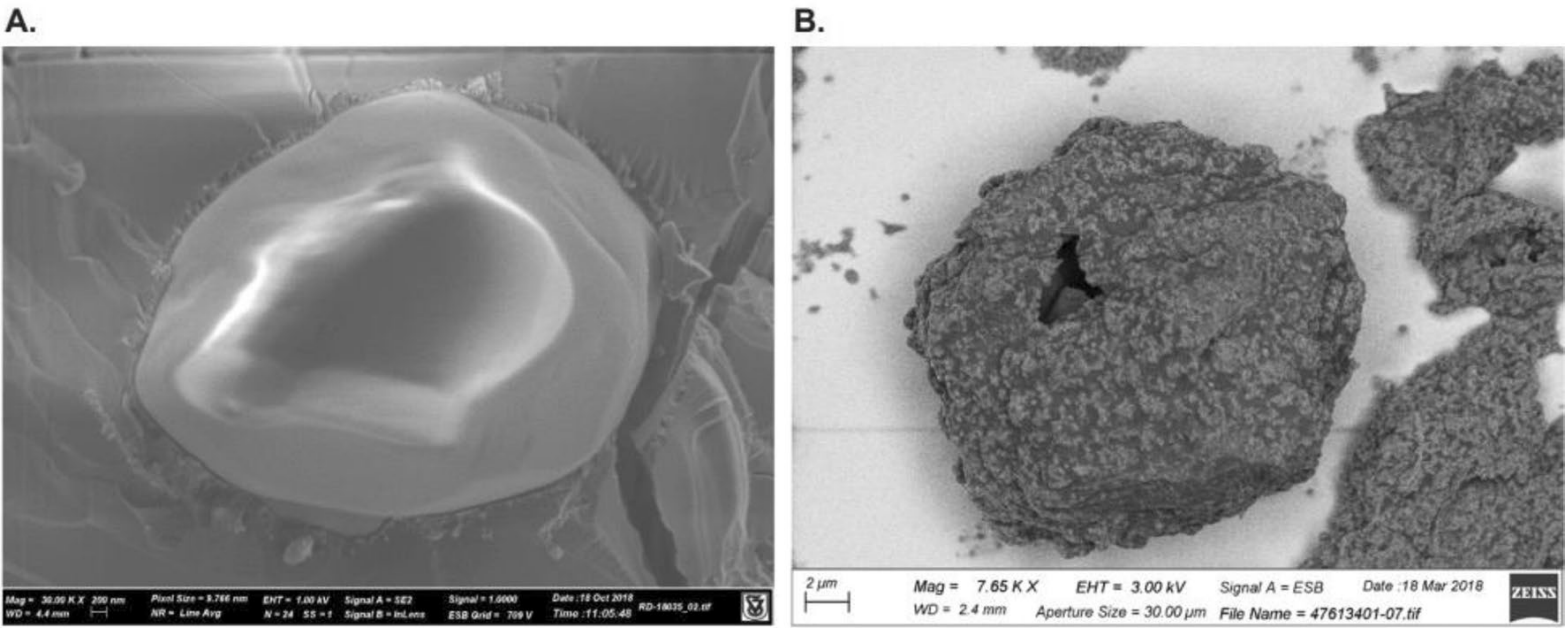
Cryo scanning electron microscopy (cryo-SEM) image of encapsulated benzoyl peroxide (E-BPO) and SEM image of encapsulated tretinoin (E-ATRA). **a** Cryo-SEM image of an E-BPO microcapsule captured with a secondary electron detector which provides morphological information. **b** SEM image captured with a back scattering electrons detector of an E-ATRA microcapsule that shows the tretinoin within the silica shell

Sol–gel microencapsulation has been successfully adapted to produce microencapsulated BPO and tretinoin. Scanning electron microscopy (SEM) and cryo-SEM images of sol–gel encapsulated all-trans-retinoic acid (E-ATRA) microcapsules indicate particle diameters ranging from 5 to 30 µm and a shell thickness of < 100 nm (Fig. 1) [25]. SEM images of sol–gel encapsulated benzoyl peroxide (E-BPO) microcapsules show particle sizes of < 30 µm, with the majority smaller than 10 µm. Shell thicknesses of the microcapsules in cryo-SEM images range from 250 to 750 nm [26]. These active ingredients are released from their microcapsules over time.

### Applications of silicon and its derivatives

Silicon (Si) is the second most copious element on earth after oxygen [27]. It is a metalloid signifying that it has both metal and nonmetal properties [28]. Si rarely occurs in its pure form and is mainly combined with oxygen (O), halogens, aluminum-forming crystalline silica (SiO_2_, quartz), amorphous silica (opal), and silicates (talc, asbestos, and mica) [29]. Silica, also known as silicon dioxide, is a silicic acid anhydride of monomeric orthosilicic acid (H_4_SiO_4_) [28]. The silicic acid group, comprised of silicon, hydrogen, and oxygen, is a group of chemical compounds with the common formula [SiO_*x*_(OH)_4−2*x*_]_*n*_ [30]_._ Metasilicic acid, orthosilicic acid, disilicic acid, trisilicic acid, and the hydrated equivalent, pyrosilicic acid, are a few simple forms identified in very dilute aqueous solutions [30]. These forms become unstable in the solid state and polymerize to form complex silicic acids [30].

**Fig. 2:**
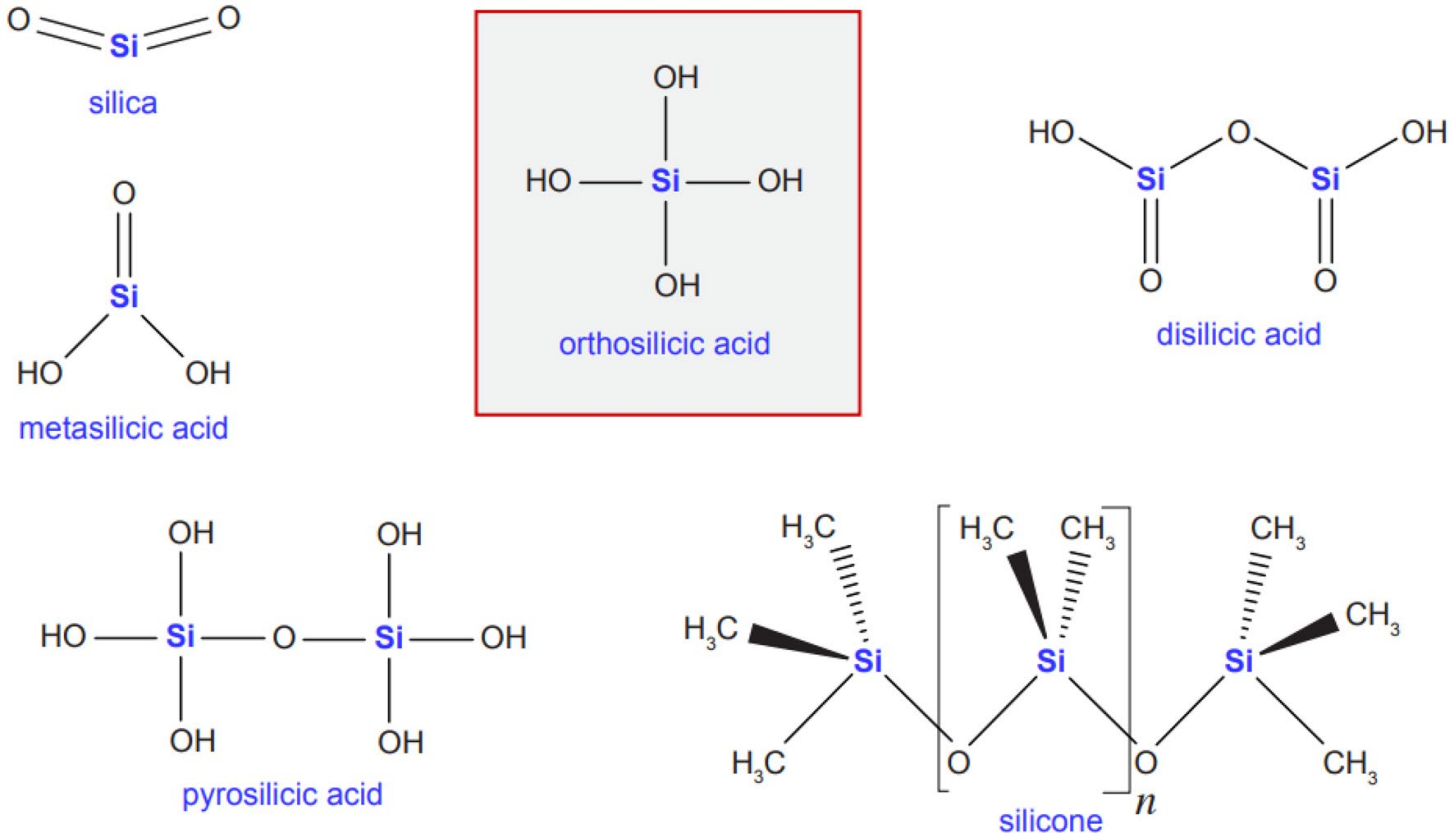
Forms of different silicic acids

Structural forms of the various silicic acids and silicone are shown in Fig. 2. Among these forms, orthosilicic acid is the most fundamental chemical form of water-soluble Si [29] and is also the natural form of Si in humans and animals [27, 29]. In the form of orthosilicic acid, Si is the third most abundant trace element in the human body [27]. Si also activates hydroxylation enzymes, enhancing skin strength and suppleness [31], and is present in 1–10 parts per million (ppm) in hair, hair epicuticle, nails, and cornified epidermis [27].

Crystalline and amorphous silica have different forms, or polymorphs, each with unique surface chemical properties. Crystalline silica is highly abrasive and used in grinding, sandblasting, and masonry projects [32]. In contrast, hydrated silica is only mildly abrasive, commonly used in toothpaste [33], and can quickly form gels that can be used in liquid foundation products [34]. Both crystalline and amorphous silica are forms of silicon dioxide which in turn is a silicic acid anhydride of monomeric orthosilicic acid.

The most common form of silica used in cosmetics and skin care products is amorphous silica [35], categorized as either natural amorphous silica or synthetic amorphous silica (SAS) [36]. Natural amorphous silica forms typically contain crystalline silica, while synthetic amorphous silica is free of crystalline silica contamination [37]. There are several forms of SAS, 2 of which include nonporous silica nanoparticles and mesoporous silica nanoparticles (MSNs) [36]. Nonporous silica nanoparticles have no particular shape or structure and have several applications due to their excellent biocompatibility. They are used in drug delivery, imaging, and enzyme encapsulation [38]. The reflective properties of synthetic amorphous silica nanoparticles (SASNs) make them excellent candidates for cosmetics and sunscreens [34, 39]. MSNs, on the other hand, have a specific structure and large surface area. Due to their well-regulated porosity and high thermal stability, MSNs are widely used in catalysis, bioimaging, and drug delivery [38]. The different silica forms can best be distinguished based on their size (Table 1) [36].

**Table 1:** Different forms of silica

Forms of silica	Particle size
Amorphous silica	
Synthetic amorphous silica	
Mesoporous silica nanoparticles	2–50 nm (controllable pore size, morphology)
Pyrogenic or fumed silica	5–50 nm
Precipitated amorphous silica	5–100 nm
Silica gel	30–100 nm
Nonporous silica nanoparticles	50–2000 nm (controllable sphere size)
Natural amorphous silica	0.5–2.0 µm
Crystalline silica	0.5–3.0 µm

Amorphous silica is an inorganic inert excipient, and the FDA currently recognizes the use of silica in the food industry as an anticaking agent [40]. According to the recent Code of Federal Regulations 21, amorphous silica is generally recognized as a safe (GRAS) ingredient in human drugs and feeds [41, 42]. The addition of amorphous silica to topical formulations may be beneficial in reducing harmful skin effects, such as irritation and rashes caused by strong active ingredients [17]. Therefore, SAS is safe when used topically, but not all forms of silica are the same, and that there are several health risks associated with crystalline silica.

Finally, silicones, not to be confused with silica, are synthetic polymers made up of repeating units of siloxane [43], elemental Si, and O combined with other elements (typically carbon [C] and hydrogen [H]) with the molecular formula of [R_2_SiO]_*n*_ (R = CH_3_, C_2_H_5,_ or C_6_H_5_). Silicones have different functional uses than silica and are commonly used as gels or sheeting to treat and minimize scars resulting from surgery, burns, and other skin injuries [44, 45].

### Uses of silica microencapsulation in topicals and sunscreens

In the current FDA inactive ingredient database (IID), which was last updated on January 2023, the maximum potency per unit dose limit for silicon dioxide used in topical creams is 3.4% w/w, and it is 0.25% w/w for topical gels [46]. Product tolerability may be improved by using sol–gel microencapsulation to coat the surface of drugs or active ingredients with a high irritation potential by reducing the contact with biological components and membranes in human skin. The FDA recently approved two new first-line topical therapies: a 5.0% microencapsulated benzoyl peroxide (E-BPO) for treating papulopustular rosacea and a fixed-dose combination of microencapsulated 3.0% BPO and 0.1% tretinoin (E-BPO/T) to treat acne [47, 48]. Despite its therapeutic properties, the use of BPO has traditionally been avoided in patients with rosacea due to the high irritation rates. E-BPO is a proprietary vehicle technology to create silica-encapsulated BPO using the sol–gel microencapsulation technique. This encapsulation forms a barrier between the drug and the skin, resulting in a gradual release and absorption of BPO, allowing for efficacy in rosacea treatment while reducing tolerability issues and adverse events [47]. The microencapsulation technology in E-BPO/T enables combining BPO and tretinoin into one product. The silica microcapsules segregate and envelop each of the active ingredients, protecting tretinoin from the oxidizing effects of BPO and releasing each active ingredient separately and gradually onto the skin [48]. Sol–gel topical products contain silica particles that are larger than typical SAS nanoparticles. The processes for encapsulating BPO and tretinoin have been described previously [5]. There is one sunscreen product that uses an advanced microencapsulation technology in which the sol–gel silica coating enhances avobenzone photostability [49]. The sol–gel-treated UV filters remain on the skin surface, and the coating provides soothing skin protection. Silica encapsulation prevents the UV filter from contacting the skin surface and, subsequently, reduces avobenzone cutaneous uptake and hypersensitivity potential [49].

### Other topical applications for microencapsulation

The sol–gel technique is also used to synthesize wound-healing products [50]. Chitosan–silica (CTS–Si) materials produced through the sol–gel process have distinctive characteristics and can function as wound-dressing agents to speed up wound healing [50]. Because of their many beneficial properties, MSNs have a broad spectrum of practical features, including combating bacterial infections [51], however commercialization may be challenging. A gentamicin-loaded MSN construct with bacterial toxin-receptive lipid bilayer surface shells protecting the bacteria-targeting peptide, UBI_29–41_, effectively targeted *Staphylococcus aureus* (*S. aureus*) in vitro and in vivo and hindered *S. aureus* growth in mouse models [52]. Also, hollow mesoporous silica nanoparticles (HMSNs) and nonporous MSNs are used to treat skin disorders [53]. An MSN assembly containing a small interfering RNA (siRNA) formulation can treat skin squamous cell carcinoma (SCC) [54]. The effectiveness of an MSN–siRNA formulation was investigated by administering siRNA topically to target the SCC transforming growth factor-beta receptor type 1 (TGFβR-1) gene in a mouse model. The results show that MSNPs comprising TGFβR-1 siRNA suppressed TGFβR-1 by twofolds compared with controls [54]. Further research is needed to test their efficacy and safety in humans.

### Silica uses in the functional design of a controlled drug delivery system

Due to their ordered mesoporous structure, functional moieties can be appended to the surface of MSNs, regulating the delivery of bioactive agents in response to different stimuli, including light, temperature, pH, electric fields, and chemicals [55]. In one study, hollow silica particles were mixed with microgels to generate novel organic/inorganic systems called thermoresponsive hollow silica microgels (THSMGs) [56]. These showed sensitivity to stimuli and might function as sustained drug delivery agents [56]. Microparticles are a unique category of drug delivery systems in which the microencapsulation technique enhances the photostability of drugs that undergo photodegradation [57]. Microencapsulation increased pantoprazole’s photostability, making the drug acid resistant and extended its release for 9 h, making it more patient compatible.

### Use of silica microencapsulation in oral medications

Oral drug delivery systems (ODDS) use silica-based materials due to their porous nature, minimal toxicity, and solubility in biological fluids [58]. The primary advantage of using silica-based drug materials in oral medications is when silica undergoes enzymatic breakdown, and the byproduct orthosilicic acid is formed, and it is then excreted by the kidneys into the urine and thought to be harmless [58, 59]. The four types of silica-based materials used in oral delivery systems are (a) nonporous silica nanoparticles (fumed or Stöber nanoparticles), (b) mesoporous silica nanoparticles (MSNs), (c) mesoporous silica-based materials, and (d) biosilica [58]. A combination of 2 MSNs, mobile composition of factor no. 41 (MCM 41) and MCM 48 were used to encapsulate aprepitant [60]. Aprepitant is an oral capsule used to prevent chemotherapy-induced and postsurgical nausea and vomiting [60]. Due to its low solubility and absorptivity, it must be administered at high doses. Microencapsulation with MCM 41 and 48 may help increase the solubility and availability of the medication at lower doses [60].

### Additional and future silica uses

Silica has many medical applications beyond the skin. Magnetic resonance imaging (MRI) contrast agents take advantage of MSNs' biosensing abilities. A gadolinium (Gd), Gd^3+^ incorporated MSN (Gd_2_O_3_@MSN) had desirable MRI contrast-augmentation properties, making it suitable for developing more precise and possibly even more focused contrast agents for molecular MRI [61]. It could also provide a real-time response for treatment results, perhaps improving the clinical value of MRI. In addition, the MSN structure comprises silica and Si–OH groups wherein the Si–O–Si systems are relatively stable, and silica breakdown is difficult under physiological conditions. These particles likely enable good loading of Gd_2_O_3_ but prevent the release of free Gd^3+^, lowering its toxic effects [61]. Another use of amorphous silica is as an ODDS based on encapsulated ciprofloxacin used to target a *Salmonella* intracellular infection [62]. The beneficial properties of silica-based nanoparticles make them promising candidates for the percutaneous delivery of anticancer drugs. A dabrafenib and trametinib drug combination was encapsulated in organosilica nanoparticles to treat mutant melanoma [63]. MSNs may be potential drug delivery agents for treating malignant nervous system tumors and Alzheimer's. Dementia associated with Alzheimer's and Parkinson's disease was treated using MSNs loaded with rivastigmine hydrogen tartrate [64]. Mo et al. demonstrated that tailored MSNs carrying anticancer drugs could circumvent the blood–brain barrier (BBB) in treating glioblastoma [65]. Another application of MSNs is in microneedle-mediated intradermal vaccination, wherein a microneedle array coated with a lipid–MSN nano construct served as an intradermal transport system for encapsulated protein antigens [66]. Silica nanoparticles can also simulate pathogen spread by contact transmission. A pilot study was performed in which silica nanoparticles in encapsulated DNA (SPED) served as a surrogate tracer to study microbial spread [67]. The pilot study results show that SPED could be a valuable and safe tool for studying pathogen propagation [67].

## Conclusion

The versatile use of amorphous silica as an excipient in healthcare and medicine has demonstrated remarkable potential in the field of drug discovery. This review has shed light on the various forms of silica and the benefits of silica microencapsulation. The use of sol–gel microencapsulation technology has enabled the development of innovative dermatological products, offering new treatments for patients suffering from conditions such as rosacea and acne. The creation of a protective silica shell between the medication and the skin has resulted in a more controlled delivery, increasing the efficiency of treatments while minimizing the adverse side effects. The continuing advancements in microencapsulation techniques have opened up new possibilities for the future of drug delivery and offer exciting opportunities for the development of novel medical treatment applications. In summary, the potential for amorphous silica and sol–gel microencapsulation technology in healthcare is enormous and requires continued research and development to explore its full capabilities.
